# Self-restraint, subsidy, and stock market reactions to the coronavirus outbreak: Evidence from the Japanese restaurant industry

**DOI:** 10.1371/journal.pone.0278876

**Published:** 2022-12-14

**Authors:** Hideaki Sakawa, Naoki Watanabel

**Affiliations:** Graduate School of Economics, Nagoya City University, Nagoya, Aichi, Japan; Universiti Malaysia Sabah, MALAYSIA

## Abstract

This study examined the stock market response of the Japanese restaurant industry to the announcement of the self-restraint request and subsidy for restaurants by the Japanese government during the coronavirus outbreak. Using the event study approach, it was found that the market reacted negatively to the self-restraint request and positively to the subsidy for restaurants. Following the announcement of the self-restraint request, investors in the restaurant industry responded positively to the government’s stringent policy responses. Conversely, following the announcement on the “dining-out” subsidy, investors reacted negatively to the stringent government policies. Our findings provide useful information for policy makers and practitioners to mitigate losses in the hospitality industry during the pandemic.

## 1. Introduction

The coronavirus disease 2019 (COVID-19) pandemic has had a massive and negative impact on the global economy [1]. COVID-19 spread rapidly worldwide, and the World Health Organization (WHO) declared it a pandemic in March 2020. There has been a sudden and sharp shortfall in firms’ revenue because the spread of COVID-19 is prominent in the hospitality industry. The Japanese government adopted self-restraint requests instead of strict lockdown restrictions for the restaurant industry [2], which affected the management of restaurants severely. Moreover, the Japanese government announced a subsidy offering a “dining-out” discount, named the *Go to Eat Campaign*, which was intended to mitigate the damage of COVID-19 on the restaurant industry [3]. This study aimed to investigate the effects of governments’ self-restraint request or subsidy for dining out in the restaurant industry.

The WHO China Country Office first detected COVID-19 in China on December 31, 2019 [4]. On January 23, 2020, the Chinese government declared lockdown in Wuhan [1]. This declaration had a negative impact on market returns in Japanese public-listed tourism firms [5]. Previous studies have implied that the announcement of a lockdown by the Chinese government caused investors in both China and Japan to be fearful of the COVID-19 pandemic. On February 3, 2020, the outbreak of COVID-19 on the cruise liner Princess Diamond was reported and the subsequent Japanese government’s policy would negatively affect the stock returns on the Japanese shipping industry [6].

On February 17, Prime Minister Abe announced the self-restraint request regarding going out in Japan. Growing fears over COVID-19 resulted in a shrinking domestic hospitality industry through government policies such as self-restraint requests and business trip warnings [7]. Therefore, there was a possibility that the self-restraint request by governments would negatively affect the stock returns in the restaurant industry because of a decrease in the number of customers. Hence, to mitigate the negative shock to the restaurant industry, the Japanese government not only declared a state of emergency, asking that citizens avoid unnecessary outings including dining out, but also announced a domestic dining subsidy on April 7, 2020 [8]. A state of emergency does neither constitute a hard lockdown nor strict restriction in Japan [9]. In this sense, this rule is not considered a heavy-handed rule imposed by the central government [10]. On the same day of declaring a state of emergency, the government implemented this subsidy and offered a “dining-out” discount to stimulate the local restaurants and dining industry [3].

This study focused on the effects of both the self-restraint request and the subsidy on the damage caused by COVID-19 by using the stock reactions of restaurant firms. Within the hospitality industry, the restaurant industry was regarded as having widely embedded and enormous business risks during the pandemic [11]. In the U.S. restaurant industry, stock returns responded negatively to COVID-19 [12]. Governments worldwide decided to implement lockdowns to restrict the spread of COVID-19 [13].

This study aimed to analyze two major events regarding the Japanese government’s policy responses related to the hospitality industry by applying an event study methodology. We first analyzed whether self-restraint requests negatively affected stock responses to COVID-19 in the restaurant industry (Hypothesis 1). We regarded the first event (Event 1) as having occurred on February 17, 2020. Self-restraint was not considered a heavy-handed rule for the Japanese residents. The second event (Event 2) ensued on April 7, 2020, when the subsidy for dining out given to local restaurants was determined and announced by the government (Hypothesis 2). On the same day, the subsidy for domestic travel was also announced. The demand for local travel has increased post the domestic travel subsidy in Japan [9].

Our study investigated whether government policy responses to COVID-19 were effective in mitigating the negative stock responses to self-restraint requests (Hypothesis 3a) and in mitigating the positive stock responses to the subsidy for dining out in the restaurant industry (Hypothesis 3b). Government interventions entail negative effects on market return of hospitality industry in the United States [14]. In addition, government policies such as a stringent distancing policy tended to negatively affect stock returns in the Japanese tourism industry [5]. This study investigated the effect of government policy on stock market returns in the restaurant industry by using market participants’ perceptions.

Various countries’ COVID-19 market responses are an important research arena in the context of restaurant industry [12], which is regarded as having a widely embedded, high level of business risk and was notably affected during the pandemic crisis [11]. The COVID-19 pandemic, might well have triggered a global economic crisis in this industry [12]. Declining stock returns post the COVID-19 pandemic have been observed in the U.S. restaurant industry [12, 15]. The COVID-19 effects on the restaurant industry in other countries have not been previously investigated and are a valid research topic [12]. In this study, we aimed to specifically provide the evidence of COVID-19 market effects in the Japanese restaurant industry.

In addition, we focused on the effects of the subsidy for dining out on local restaurants. On April 7, 2020, the Japanese government announced the domestic subsidy to mitigate the negative effects of COVID-19. In fact, the domestic subsidy contributed to enhancing demand for local travel in Japan [9]. Kanamori et al. [3] pointed out that this subsidy led to an increase in COVID-19 cases owing to increased mobility by changing behavior. So far, previous studies have not investigated the effects of these subsidies on Japanese restaurant firms.

Using publicly listed restaurant firms in Japan, this study presents several findings by applying an event study methodology [1, 14]. First, we found that the uncertainty of COVID-19 negatively affected stock market returns in the restaurant industry, which was consistent with Song et al.’s [12] findings. The self-restraint requests triggered by COVID-19 had a negative effect on stock returns. Second, this study revealed that the dining-out subsidies served to mitigate the negative impact of the self-restraint requests during COVID-19. This result implied that the Japanese government’s stimulus was beneficial in supporting the restaurant industry. Third, as for Event 1, government and social distancing policies positively affected stock returns in the Japanese restaurant industry. We may infer that these policies helped in mitigating the negative effect of COVID-19 uncertainty. Finally, we found that for Event 2, government and social distancing policies reduced the positive effect of the stimulus for local restaurants.

Our study contributes to the literature in several ways. First, it adds to the overall understanding of COVID-19 damages to the restaurant industry [12] by showing how the self-restraint request and the subsidy for local restaurants regarding COVID-19 affected investor sentiments. This study adds to the findings of Matsuura and Saito [9] by highlighting the role of the dining-out subsidy in enhancing the stock market reaction. Second, this study provides an insight into both the self-restraint request and subsidy for restaurants under severe government policy responses to COVID-19 as well as its perception of market participants. Government policy responses, such as the semi-closure of the tourism industry, affect the negative impact of COVID-19 on tourism stocks [16]. In the restaurant industry, the uncertainty of COVID-19 negatively affected the stock market returns [12].

## 2. Methodology and research design

### 2.1. Background and methodology

Previous studies have investigated how COVID-19 affects the stock return in hospitability industry including restaurants, hotels, and tourism. The stock prices of listed hotels have been negatively affected by the COVID-19 outbreak [17]. Jawed et al. [18] investigated the negative impact of COVID-19 on stock returns for hospitality and tourism firms in India using an event study method (ESM). The U.S. restaurant industry has negatively responded to the COVID-19 outbreak [12]. Thus, stock prices of Japanese restaurant firms may have been negatively affected by the COVID-19 outbreak. By contrast, previous studies did not fully analyze the pandemic-recovery-based stimulus subsidy effect [19]. During the COVID-19 pandemic, stimulus packages such as “dining out subsidy” have been shown to be positively related to market returns [20]. As the stimulus packages would recover the demands of customers in the restaurants, the sentiments of investors in the restaurant industry would be positive. Thus, we conjecture that the announcement of the dining-out subsidy positively affected publicly listed Japanese restaurant firms.

This study aimed to analyze whether the COVID-19 outbreak impacted stock returns in the restaurant industry in Japan. As mentioned, Prime Minister Abe encouraged Japanese residents to stay home voluntarily on February 17, 2020. On April 7, 2020, the Japanese government announced a dining-out discount campaign to minimize the damage COVID-19 inflicted on the restaurant industry. This campaign aimed to invigorate the restaurant industry.

There are two reasons why we focused on these two events. To clarify this point, we use the additional table which shows the timeline of COVID-19 related news around the date of Events 1 and 2. Event 1 is thought of as the first negative news which directly implicated the Japanese restaurant industry. On January 30, WHO declared a “public health emergency.” However, there may have been no direct measures to affect Japanese corporations before the event 1 (February 14th). Next, we focus on Event 2 (“Go-to-campaign”) which entails the positive effects for the stock returns of restaurant industry. Certainly, other types of “negative event” have subsequently occurred post Events 1 and 2. We presumed that if the announcement effect of Event 2 was weak, its positive effects would not have persisted. The timeline is shown in A1 Appendix in S1 File.

Following Aharon et al. [14] and Poretti and Heo [21], we adopted an ESM to investigate the stock price movements in Japan. We set the two event dates as February 17, 2020, and April 7, 2020, respectively (day = 0). This study implemented the ESM to analyze stock market reactions to estimate abnormal returns (AR) using the market model [13, 22]. We set the estimation window as 120 to 21 days prior to the event date (-120, -21) [23, 24]

$$R_{it}=\beta_{0}+\beta_{1}R_{mt}+\varepsilon_{it}$$

$$where\;E[\varepsilon_{it}]=0,Var[\varepsilon_{it}]=\sigma_{it},$$ (1)

Here, *R*_*it*_ represents the daily closing stock return at time *t*, and *R*_mt_ is the daily stock return on a value-weighted market index for Japan. Thereafter, we defined AR as each firm’s stock return minus the expected return over a given day.

We estimated the parameters applied to market returns [25] and cumulative abnormal returns (CAR) as the sum of AR over a given event window ([0, +T]). CAR are defined as in Eq (2):

$$C{AR}_{i}[t]=\sum_{t=0}^{T}{AR}_{it}.$$ (2)

We checked the significance of AR and the CAR by using the t-test. The effectiveness of an event was confirmed if the values of CAR significantly differ from 0.

### 2.2. Hypotheses development

The high level of business risk during the pandemic crisis negatively affected the restaurant industry [11]. The negative stock returns due to the COVID-19 crisis has been investigated in the U.S. restaurant industry [12, 15]. In the Japanese restaurant industry, Prime Minister Abe encouraged Japanese residents to obey a self-restraint request on February 17, 2020. A “self-restraint-request” was a less stringent measure than that of an imposed lockdown which was implemented in other countries such as the U.S. The “self-restraint-request” seemed to induce anxiety in people as it created uncertainty [26], reducing demand on restaurants. We inferred from this that the Japanese government’s “self-restraint-request” would induce the negative sentiment of investors and the subsequent negative stock return. Therefore, we present the following Hypothesis 1.

### Hypothesis 1

Self-restraint requests negatively affected stock responses to COVID-19 in the restaurant industry.

On April 7, 2020, Japanese government announced the subsidy for “dining-out” discount, named the *Go to Eat Campaign*, to mitigate the damage of COVID-19 on the restaurant industry [3]. Owing to this campaign, the stock price of restaurant industry was predicted to rise because the decreasing demand of restaurant industry might be mitigated. Thus, we present the following Hypothesis 2.

### Hypothesis 2

The subsidy for dining out given to local restaurants positively affected stock responses to COVID-19 in the restaurant industry.

Government responded to the COVID-19 outbreak by implementing several measures such as stringent distancing policies [16]. The sentiment of anxiety due to the “self-restraint requests” would be mitigated by government measures such as stringent distancing policies. Thus, we present the following Hypothesis 3a.

### Hypothesis 3a

Stricter government policy responses to COVID-19 were effective in decreasing the negative stock responses to self-restraint requests in the restaurant industry.

As for the announcement on “Go-to-Eat” campaign, the positive effect of this subsidy would be mitigated if government and social distancing policies are strengthened. During the COVID-19 outbreak, the sales of restaurants decreased due to strict government measures. In Chinese restaurants, the decrease of sales in regions with extensive shutdowns was greater than those with less strict ones [27]. Thus, stricter measures would decrease the demand of the restaurant industry and mitigate the positive effect of the campaign to the stock return. Therefore, we constructed the following hypothesis 3b.

### Hypothesis 3b (Go-to-Eat)

Government policy responses to COVID-19 were effective in reducing the positive stock responses in the restaurant industry.

### 2.3. Research design and data

We investigated how the government response to the COVID-19 outbreak influenced the announced domestic dining-out subsidy’s effect on Japanese restaurant stocks. To protect the lives and health of the people, the Japanese government implemented several measures to restrict the restaurant industry. Numerous measures were also reinforced to address the threat of the pandemic. Following Al-Awadhi et al. [28] and Sakawa and Watanabel [5], we investigated the impact of government response on stock returns as follows:

$${AR}_{it}=\alpha_{0}+\beta_{1}{\left(GRI/SI\right)}_{it-1}+\sum_{j}\gamma_{j}{Control}_{it-1}+\varepsilon_{it}$$ (3)

Here, the government response index (GRI) and government stringency index (SI) were obtained from the *OxCGRT* database of several policy responses that governments have taken to respond to COVID-19 [29]. The market reaction to COVID-19 threats is higher in larger firms with more growth opportunities [12, 16]. Thus, we controlled for firm size (SIZE) as the logarithm of market capitalization [12] and the firm’s growth opportunity as the price-to-book ratio (PBR) [16]. As cashflow of a firm is a good predictor of a firm’s capacity to deal with external shocks, including COVID-19, we also controlled for the liquidity of firms, using cash to assets (CASH) [12] and financial conditions such as liquidity of a firm, measured by a debt-to-equity ratio (Leverage) [12, 30]. Institutional ownership (INST) was also controlled for as trustworthy indicators for shareholders in sustaining their shareholding [12].

We defined the restaurant industry as firms classified under the North American Industry Classification System codes (NAICS codes) 722511 and 722513 [12]. We collected financial data and daily stock returns from the *Astra Manager* provided by *Quick* and *NPM database* provided by *Financial Data Solutions*, respectively. The number of our sample is 93. We excluded firms with missing data of institutional ownership during the event window. Next, we gathered data on the number of confirmed COVID-19 cases and deaths from the website of the Ministry of Health, Labour, and Welfare [31] and data based on government indices from the *OxCGRT* website. The data definitions are summarized in A2 Appendix in S1 File.

## 3. Empirical results

### 3.1. Descriptive statistics

We introduced descriptive statistics of the variables in Table 1. Panels A and B show descriptive statistics of Event 1 and Event 2, respectively. The mean value of AR was -0.762 in Event 1 and 0.616 in Event 2. The average value of daily growth of COVID-19 cases was about 9.2% in Event 1 and 3.4% in Event 2. The average PBR and Leverage were about 8.39 and 0.54, respectively.

**Table 1:** Results of AR.

	Event 1 (February 17^th^)			Event 2 (April 7^th^)		
Event window	AR		t value	AR		t value
0	-1.79	**	(-9.03)	2.53	**	(6.92)
1	-1.27	**	(-7.21)	3.71	**	(10.85)
2	0.68	**	(5.29)	0.96	*	(2.30)
3	-0.51	**	(-4.18)	-1.12	*	(-2.35)
4	-0.17		(-0.95)	0.71	*	(2.16)
5	-1.88	**	(-9.27)	-0.61	**	(-2.65)
6	-1.37	**	(-6.80)	0.57	*	(2.12)
7	-3.78	**	(-9.92)	1.17	**	(5.67)
8	-6.03	**	(-17.71)	-0.42		(-1.56)
9	4.83	**	(14.39)	1.73	**	(8.79)
10	-0.86	**	(-3.09)	-0.25		(-1.27)
11	-0.26		(-1.32)	-1.23	**	(-5.22)
12	0.13		(0.69)	-0.14		(-0.72)
13	-2.28	**	(-10.35)	-0.27		(-1.56)
14	-4.40	**	(-13.59)	-0.61	*	(-2.56)
15	0.41		(0.97)	1.64	**	(6.48)
16	-0.81	**	(-2.84)	0.35	+	(1.75)
17	-3.92	**	(-11.48)	1.38	**	(8.04)
18	-5.38	**	(-13.59)	0.13		(0.65)
19	1.67	**	(4.22)	1.84	**	(4.99)
20	2.10	**	(4.02)	3.72	**	(9.49)
21	3.06	**	(7.02)	0.25		(0.75)
22	1.19	*	(2.16)	0.12		(0.73)
23	1.57	**	(4.45)	0.27		(0.90)
24	2.26	**	(5.19)	-0.82	**	(-4.07)
25	2.05	**	(5.69)	0.33		(1.04)
26	-2.72	**	(-6.69)	-0.82	**	(-3.81)
27	-1.45	**	(-4.82)	0.73	*	(2.03)
28	-4.88	**	(-9.78)	1.27	**	(3.19)
29	1.03	**	(4.13)	0.78	**	(3.36)
30	-2.31	**	(-7.90)	1.15	**	(3.12)
Number	93			93		

AR stands for abnormal returns. **, *, and+ are significant at 1%, 5%, and 10% confidence levels, respectively. + p<0.10, * p<0.05, ** p<0.01.

### 3.2. Market response

We show the market responses on the two announcements to provide the average of AR (AAR) in Table 2. For Event 1, we observed 10 positively significant and 17 negatively significant AAR during the post-event window. This suggests that the government’s self-restraint request was detrimental to the Japanese restaurant industry. By contrast, we found 15 positively significant and 6 negatively significant AAR during the post-event window for Event 2. This implies that investors evaluated the dining-out subsidy as advantageous for restaurants.

**Table 2:** Results of CAR (cumulative abnormal return).

	February 17^th^			April 7th		
Event window	CAR		t value	CAR		t value
0 / +3	-2.89	**	(-9.90)	6.08	**	(9.59)
0 / +5	-4.94	**	(-10.90)	6.17	**	(8.72)
0 / +8	-16.11	**	(-17.90)	7.49	**	(9.18)
0 / +10	-12.13	**	(-14.20)	8.98	**	(11.70)
0 / +20	-24.87	**	(-12.32)	15.79	**	(12.32)
0 / +30	-25.07	**	(-12.90)	19.05	**	(10.96)
Number of Firms	93			93		

CAR stands for cumulative abnormal returns. **, *, and+ are significant at 1%, 5%, and 10% confidence levels, respectively. + p<0.10, * p<0.05, ** p<0.01.

Table 3 shows the results of the average of the CAR from the post-event window. Negative and significant CAR remained after 30 trading days in Event 1 (February 17). This result indicates that the negative announcement effects of the self-restraint request by governments continue for more than 1 month in restaurant industry, consistent with Hypothesis 1. As for Event 2 (April 7), we find significant and positive CAR post 30 trading days, supporting our Hypothesis 2. These results show that the announcement of the domestic subsidy had a positive effect on Japanese restaurant firms for a relatively long period.

**Table 3:** Descriptive statistics.

Panel A. Descriptive Statistics of Event 1						
Variable	Number	Mean	SD	Q1	Median	Q3
AR	2697	-0.762	3.985	-2.749	-0.710	1.163
Case	2697	0.092	0.042	0.057	0.091	0.129
GRI	2697	0.032	0.100	0.000	0.000	0.000
SI	2697	0.687	2.512	0.000	0.000	0.000
Size	2697	5.262	1.553	4.096	5.382	6.434
PBR	2697	8.389	34.331	1.801	3.015	5.272
Cash	2697	0.070	0.052	0.040	0.078	0.104
Institution	2697	5.631	5.351	0.600	4.760	10.100
Leverage	2697	0.544	0.209	0.372	0.558	0.712
Panel B. Descriptive Statistics of Event 2						
Variable	Number	Mean	SD	Q1	Median	Q3
AR	2697	0.616	3.017	-0.941	0.312	1.747
Case	2697	0.034	0.038	0.003	0.018	0.061
GRI	2697	-0.001	0.034	0.000	0.000	0.000
SI	2697	-0.388	2.047	0.000	0.000	0.000
Size	2697	5.262	1.553	4.096	5.382	6.434
PBR	2697	8.389	34.331	1.801	3.015	5.272
Cash	2697	0.070	0.052	0.040	0.078	0.104
Institution	2697	5.631	5.351	0.600	4.760	10.100
Leverage	2697	0.544	0.209	0.372	0.558	0.712

See A2 Appendix in S1 File for definitions and measurements of the variables.

**Fig 1:**
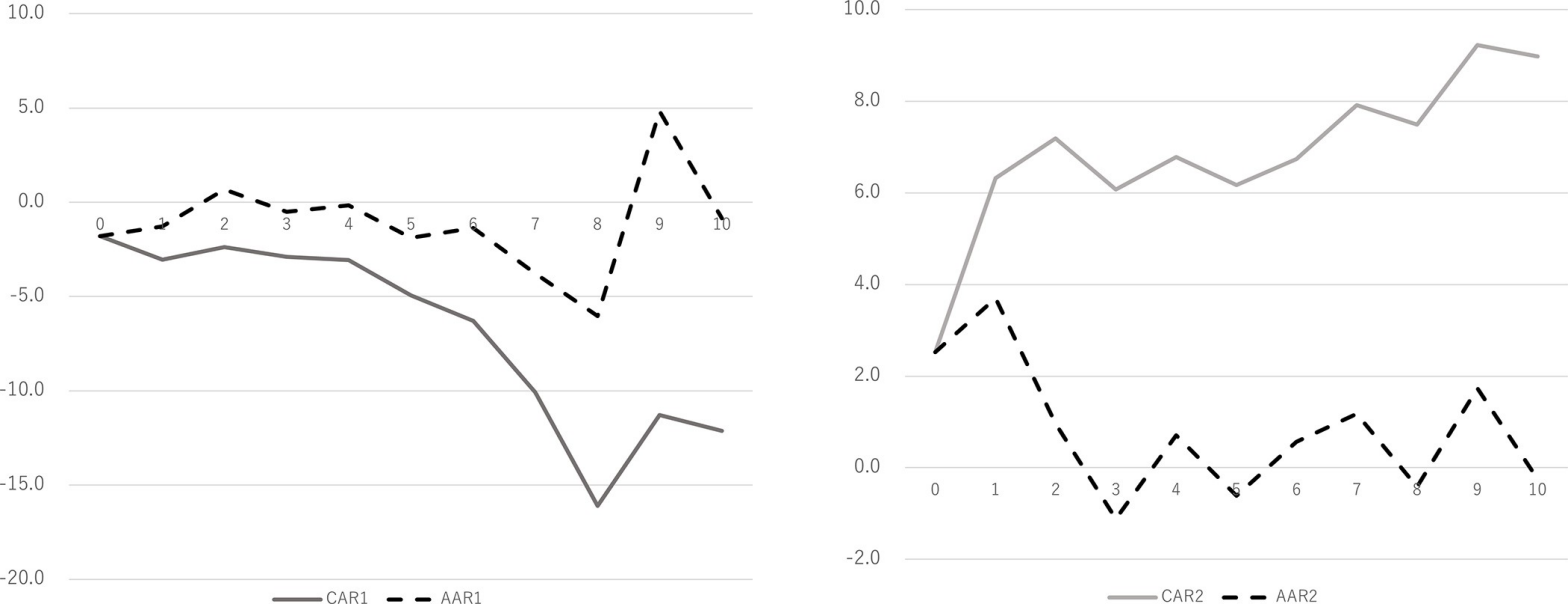
CAR and AAR. A: This is the Panel 1 Fig 1 (Panel 1. CAR and AAR during the event 1). B: This is the Panel 2 Fig 1 (Panel 2. CAR and AAR during the event 2). Note. CAR stands for cumulative abnormal returns. AAR stands for the average of abnormal returns.

By comparison, Panel A and Panel B of Fig 1 show each of the AAR and the average of CAR for the two events: Event 1 and 2. Using Panel A of Fig 1, we found that CAR persisted for negative values post Event 1, suggesting that the self-restraint request was not favored by market participants in the short term, which is consistent with lockdown evidence from India [13]. Panel B of Fig 1 indicates a gradually increasing tendency of CAR post Event 2, implying that the subsidy announcement was considered favorable for the restaurant industry given the self-restraint request because the subsidy would mitigate the effects of decreasing demands under the self-restraint request in Japanese restaurant industry.

**Fig 2:**
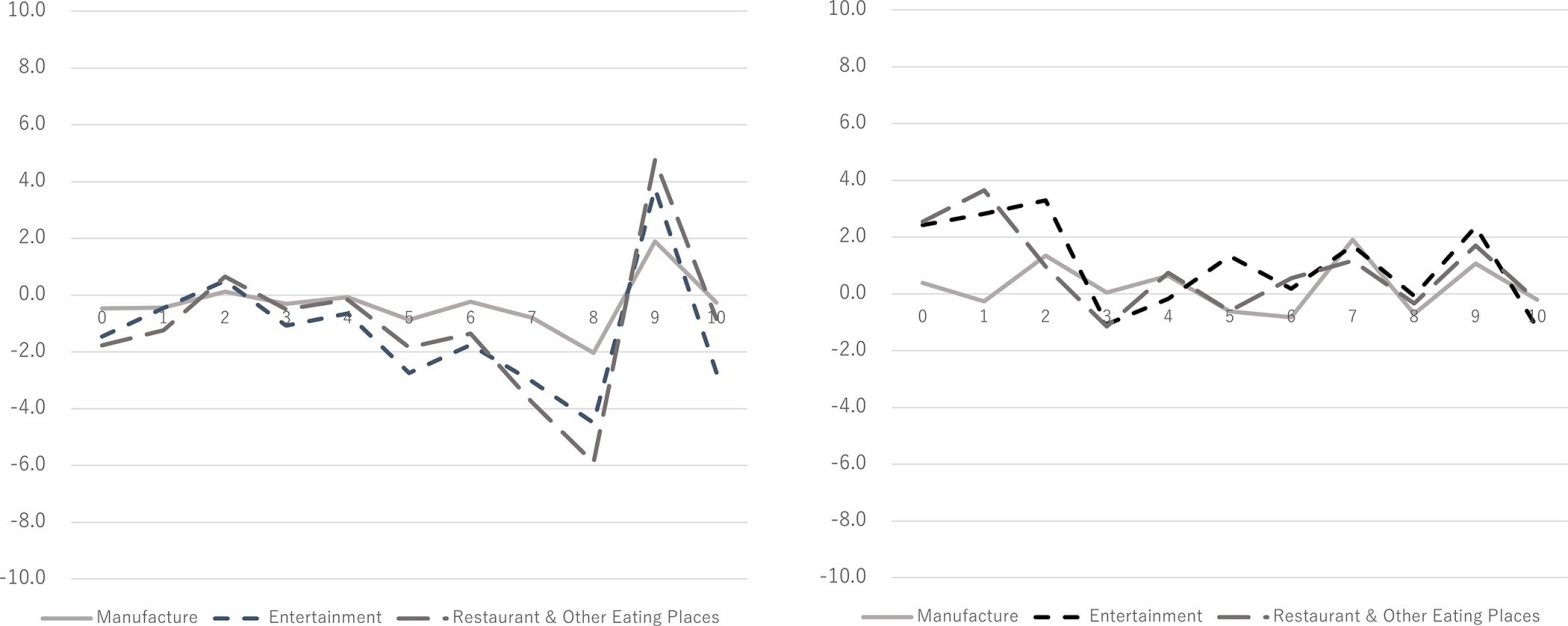
AAR during the two events. A: This is the Panel 1 Fig 2 (Panel 1. February 17th). B: This is the Panel 2 Fig 2 (Panel 2. April 7th). Note. AAR stands for the average of abnormal returns.

To compare the stock patterns of restaurant firms with other related industries affected by the “Go-to-Eat” campaign, we chose the entertainment industry whose NAICS code is 71. In addition, we also chose the manufacturing industry firms classified by NAICS codes 31 and 33, which were not affected by the campaign. To consider the difference within the restaurant industry, we additionally check the restaurants and other eating places whose NAICS code is 7225. From Panel A of Fig 2, we find that the stock pattern of manufacturers is different from those of the restaurant and entertainment industries. Panel B of Fig 2 also indicates that the stock pattern of manufacture differs from those in other industries such as restaurant and entertainment. Thus, we confirmed that the “Go-to-eat” campaign affected only industries which were the target of government subsidies.

**Fig 3:**
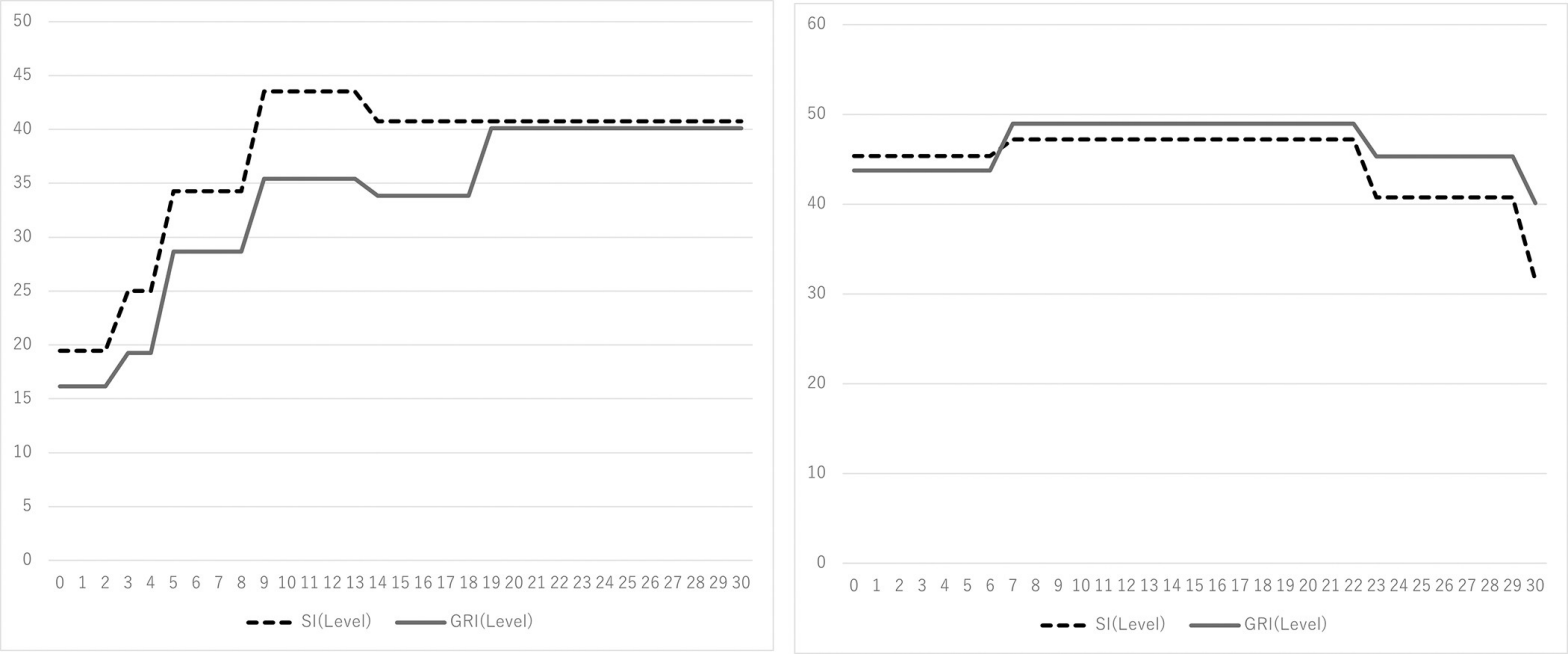
SI and GRI during the two events. A: This is the Panel 1 Fig 3 (Panel 1. February 17th). B: This is the Panel 2 Fig 3 (Panel 2. April 7th). Note. See A2 Appendix in S1 File for definitions and measurements of the variables.

We also summarize the patterns of Stringency index (SI) and Government Response index (GRI) after two events in Fig 3. From Panel A, we find that both SI and GRI increased post Event 1. From Panel B, we find both of GMI and SI have increased during the post 23 trading days of the Event 2 (May 14^th^). After May 14, the state of emergency was lifted in 39 prefectures and the level of GMI and SI has decreased. Thus, these two indexes are connected to the COVID-19 situation in Japan.

### 3.3. Estimated results

We show the estimated results of Eq (2), which analyzed the government’s response to stock returns, in Table 4. For Event 1, we found that *GRI* (*SI*) was significantly positive for the AR in Columns (1) and (2). This suggests that the stringent government policy responses to the COVID-19 outbreak was effective in mitigating negative market reaction, consistent with non-pharmaceutical intervention effects [32]. We also found that the interaction terms of *GRI* (*SI*) and *Case* were significantly negative, which suggests that the positive announcement effects on *GRI* (*SI*) were negatively moderated by the increase in COVID-19 cases, supporting our Hypothesis 3a.

**Table 4:** Estimated results.

	(1)		(2)		(3)		(4)		(5)		(6)		(7)		(8)	
	February 17th				April 7th				February 17th				April 7th			
	Abnormal Return															
Case	-10.890	**	-10.910	**	2.835		1.645									
	(-7.28)		(-8.10)		(1.60)		(0.88)									
Lag_Case									-16.070	**	-17.790	**	1.699		0.914	
									(-18.18)		(-20.21)		(0.94)		(0.49)	
GRI	4.492	**			-6.698	**			28.450	**			-5.728	*		
	(3.82)				(-2.66)				(10.36)				(-2.28)			
GRI	-28.660	+			259.300	**										
* Case	(-1.90)				(4.99)											
GRI									-177.900	**			278.900	**		
* Lag_Case									(-9.13)				(5.06)			
SI			0.347	**			-0.086	**			1.218	**			-0.084	**
			(9.86)				(-2.80)				(10.30)				(-2.75)	
SI			-1.425	**			10.640	**								
* Case			(-3.85)				(5.18)									
SI											-6.905	**			13.030	**
* Lag_Case											(-8.09)				(5.63)	
Size	0.003		0.003		0.031		0.031		0.003		0.003		0.031		0.031	
	(0.06)		(0.06)		(0.71)		(0.71)		(0.06)		(0.06)		(0.71)		(0.71)	
PBR	0.000		0.000		0.000		0.000		0.000		0.000		0.000		0.000	
	(0.35)		(0.35)		(0.24)		(0.24)		(0.35)		(0.35)		(0.24)		(0.24)	
Cash	-2.412	+	-2.412	+	2.361	+	2.361	+	-2.412	+	-2.412	+	2.361	+	2.361	+
	(-1.80)		(-1.80)		(1.75)		(1.75)		(-1.80)		(-1.80)		(1.75)		(1.75)	
Institution	0.020		0.020		0.009		0.009		0.020		0.020		0.009		0.009	
	(1.61)		(1.61)		(1.05)		(1.05)		(1.61)		(1.61)		(1.05)		(1.05)	
Leverage	-0.767	**	-0.767	**	0.300		0.300		-0.767	**	-0.767	**	0.300		0.300	
	(-2.73)		(-2.73)		(1.13)		(1.13)		(-2.73)		(-2.73)		(1.13)		(1.13)	
Constant	0.610	+	0.534	+	-0.136		-0.108		1.073	**	1.219	**	-0.093		-0.087	
	(1.90)		(1.69)		(-0.42)		(-0.33)		(3.40)		(3.89)		(-0.29)		(-0.27)	
Number	2697		2697		2697		2697		2697		2697		2697		2697	
Adjusted R2	0.023		0.043		0.013		0.017		0.093		0.114		0.010		0.016	
F	22.61	**	34.12	**	6.66	**	6.55	**	71.83	**	72.10	**	6.59	**	6.63	**

This table shows regression results of the growth in COVID-19 confirmed cases and abnormal returns from days 0 to 30. Lag_Case means a lag variable of the Case. Standard errors are clustered by firm. t value in parentheses. **, *, and+ are significant at 1%, 5%, and 10% confidence levels, respectively. + p<0.10, * p<0.05, ** p<0.01.

As for Event 2, we found that *GRI* (*SI)* is significantly negative for the AR in Columns (3) and (4). This implies that the stimulus effect for local restaurants was restricted to a great degree by the stringent government policy responses to the COVID-19 outbreak, supporting our Hypothesis 3b. In addition, we also found that the cross terms of *GRI* (*SI*) and *Case* were significant and positive. This implies that the negative announcement effects on *GRI* (*SI*) were positively moderated by the increase in COVID-19 cases.

Overall, these results prove that the mitigation of the outbreak through the implementation of social distancing policies was directly linked to stock returns, especially for the restaurant industry. Considering the fact that the previous day’s number of confirmed cases was reported by Japanese medias, we also check whether our results are robust when we adopted the lagged variables of *Case* (*Lag Case*) in Columns (5), (6), (7), and (8) of Table 4. The estimated results are similar and, therefore, confirmed the robustness of the results.

Regarding the control variables, *LEVERAGE* is significantly negative for the AR in Columns (1), (2), (5), and (6) suggesting that the higher COVID-19-related business risk resulted in higher investor anxiety for restaurant firms post the self-restraint request [12, 33]. In addition, these negative relationships are not observed for Event 2. The other control variables are not significant at the 5% level.

## 4. Conclusions

This study evaluated the impact of the COVID-19 outbreak on Japanese restaurant stocks. First, we found that the Japanese government’s announcement of the self-restraint request had a negative effect on the restaurant stocks, implying that the request related to COVID-19 negatively affected the Japanese restaurant industry. To prevent the spread of COVID-19, Japanese government chose “Self-restraint-request”, different from strict lock-down in other countries such as U.S. and China. During the lockdown period of March to September, 2020, the negative stock returns were investigated in the U.S. restaurant industry [12, 15]. We found that the negative stock return is larger post the government’s self-restraint request in the Japanese restaurant industry, consistent with our Hypothesis 1.

In addition, the study showed that the announcement of the domestic dining-out subsidy had a positive effect on restaurant stocks. We may infer that market investors understood that the domestic dining-out subsidy was an appropriate measure for the restaurant industry’s recovery in Japan, and this is consistent with the findings of previous studies [9, 34]. We found that the negative stock return is mitigated by the domestic dining-out subsidy, consistent with our Hypothesis 2. Our study could provide further economic insight into the effectiveness of the stimulus package to recover the stock market returns during the pandemic. The effectiveness of stimulus package to the stock market returns was confirmed in the U.S. markets during COVID-19 [19]. In this study, we extended investigations into stimulus packages to that in the Japanese restaurant industry.

Furthermore, we analyzed whether the government’s response to the influence of the COVID-19 pandemic influenced stock returns. Our results revealed that social distancing and the government’s policy responses positively affected the AR post Event 1, consistent with the market responses to government intervention [29] and supporting our Hypothesis 3a. We also revealed that social distancing and the government’s policy responses negatively affected the AR of Japanese restaurant stocks post Event 2, consistent with a previous study conducted in China [16]. This result also supports our Hypothesis 3b. Finally, the positive announcement effects on social distancing and government policy responses were negatively moderated by the increase in COVID-19 cases, whereas the negative announcement effects on social distancing and government policy responses were positively moderated by the increase in COVID-19 cases.

Overall, these results contributed to the stock market responses toward COVID-19 in the hospitality industry. We showed that the market responded negatively to the self-restraint request and positively to the dining-out subsidy for restaurant firms. These results suggest that the subsidy for local restaurants was effective in mitigating the negative impact of COVID-19 under the voluntary lockdown. Government stimulus, such as the dining-out subsidy, may be useful in supporting restaurants in the early stages of a pandemic. Furthermore, the social distancing policies negatively affected the market response following the announcement of the dining-out subsidy. We may interpret that the social distancing policies especially hurt the performance of the restaurant industry. Confirmed COVID-19 cases and deaths, as well as different stock market responses to the pandemic, are important for the restaurant industry.

As for the limitations, first, since this study focused on short-term stock market responses, future studies should investigate the long-term effects of the pandemic on restaurant firms. Second, future studies should analyze the effect of stimulus packages provided by different governments during the peak of the COVID-19 crisis.

This study opens new avenues for future research on the effects of pandemics on the restaurant industry. First, the level of lockdown have varied over time in many countries. It is possible that the effects of lockdown would be differently characterized in other countries. Second, government responses to COVID-19 might change. Thus, longer-term effects of government responses are unknown. Our study can form the basis of future research in these aspects. Future research could add to the knowledge of these varying effects.

## Supporting information

S1 File (DOCX)
