# Supplementary material for: Self-restraint, subsidy, and stock market reactions to the coronavirus outbreak: Evidence from the Japanese restaurant industry
Source: PLoS One. 2022 Dec 14;17(12):e0278876. doi: 10.1371/journal.pone.0278876 (PMC9749993; doi:10.1371/journal.pone.0278876)
Supplement: S1 File — (DOCX) [file pone.0278876.s001.docx]

**Appendix A1. Timeline of Covid-19 related news around the date of Event 1 and 2**

| Date, Time | News | The predicted effects |
| --- | --- | --- |
| January 30, 2020 | Public health emergency (WHO) | Negative to the market |
| February 3, 2020 | The outbreak of COVID-19 on the Princess Diamond was reported | Negative to the market |
| February 17, 2020 | Self-restraint-request (Event 1) | Negative to the market |
| March 11, 2020 | WHO declared COVID-19 as a pandemic | Negative to the market |
| March 24, 2020 | Tokyo 2020 Olympic Game has been postponed due to the COVID-19 | Negative to the market |
| April 7, 2020 | (1) Announcement on Go-to-Campaign (Event 2) | Positive to the market in the Restaurant & Event industries |
|  | (2) A State of Emergency has been declared to the 7 prefectures in Japan | Negative to the market |
| April 16, 2020 | A State of Emergency has been declared to all prefectures (to May 6th) | Negative to the market |
| May 4, 2020 | Extend a state of Emergency to May 31th | Negative to the market |
| May 14, 2020 | A state of emergency has been lifted to 39 prefectures | Positive to the market |
| May 25, 2020 | A state of Emergency has been lifted to all prefectures | Positive to the market |

**Appendix A2.** Variable definitions

| Variable | Definition |
| --- | --- |
| AR | Abnormal returns |
| Case | The daily growth rate of COVID-19 confirmed cases calculated as  ((Case(t)-Case(t-1))/Case(t-1) |
| Lag_Case | A lag variable of the Case |
| GRI | The daily growth rate of Government response index calculated as  ((GRI(t)-GRI(t-1))/GRI(t-1) |
| SI | The daily difference of Government response stringency index calculated as ((SI(t)-SI(t-1)) |
| Size | Logarithm of market capitalization |
| PBR | The price-to- book ratio. |
| Cash | Cash flows from operating activities and investing activities divided by total assets |
| Institution | Percentage of shareholding by institutional investors |
| Leverage | Ratio of debt to total asset |
